# Supplementary material for: ChIP-Seq-Based Approach in Mouse Enteric Precursor Cells Reveals New Potential Genes with a Role in Enteric Nervous System Development and Hirschsprung Disease
Source: Int J Mol Sci. 2020 Nov 28;21(23):9061. doi: 10.3390/ijms21239061 (PMC7730166; doi:10.3390/ijms21239061)

Supplementary Material

Table S1. Additional genes selected because of their interaction with more than one of the PAX6 target genes showed by IPA tool.

		**Target genes**											
**Related genes**		***ABI3BP***	***ACADM***	***ATXN1***	***BDR3***	***COL4A2***	***DGKI***	***GNG4***	***IL10RA***	***KCNQ1***	***MCC***	***RABGGTB***	***SORBS1***
	TGFB1			X		X			X		X		
	VHL					X	X	X			X		
	APP			X	X						X		
	MYC				X	X							X
	NTRK1		X		X								X
	GRB2	X								X	X		
	HRAS			X								X	X
	HTT		X			X							X
	TNF					X			X				X
	HIST1H3A			X	X								

Figure S1. Average Ct values of candidate genes expressed in NLBs cultures from human. The upper limit of the Ct was set to be 35.

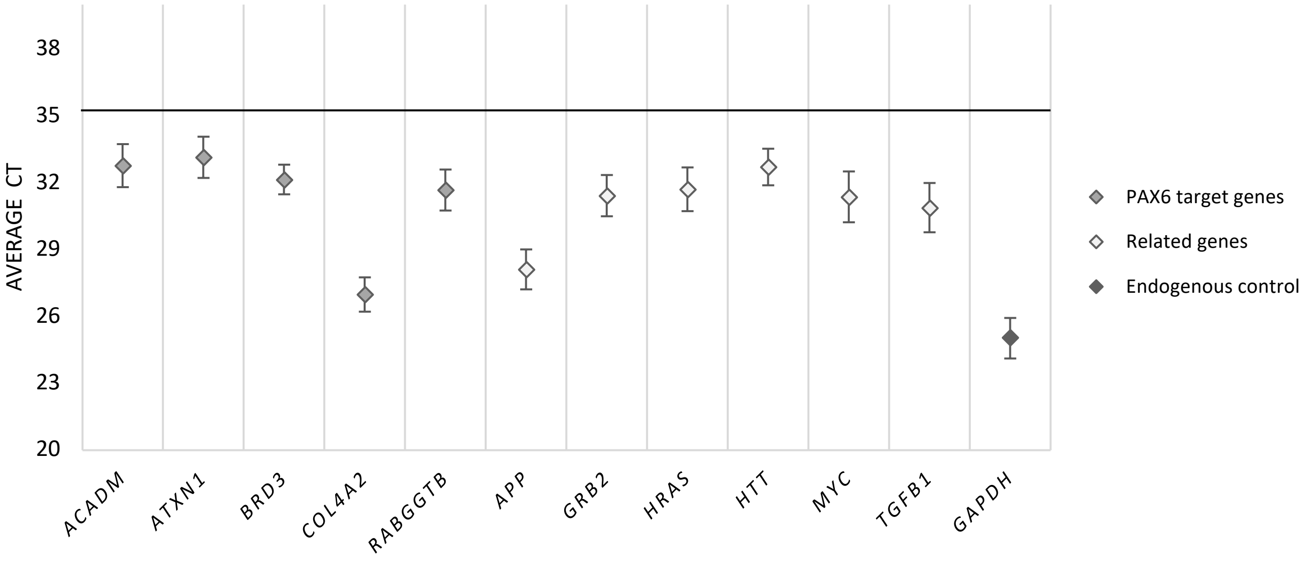

Figure S2. Functional networks shared among the potential susceptibility genes for HSCR provided by GeneMANIA database.

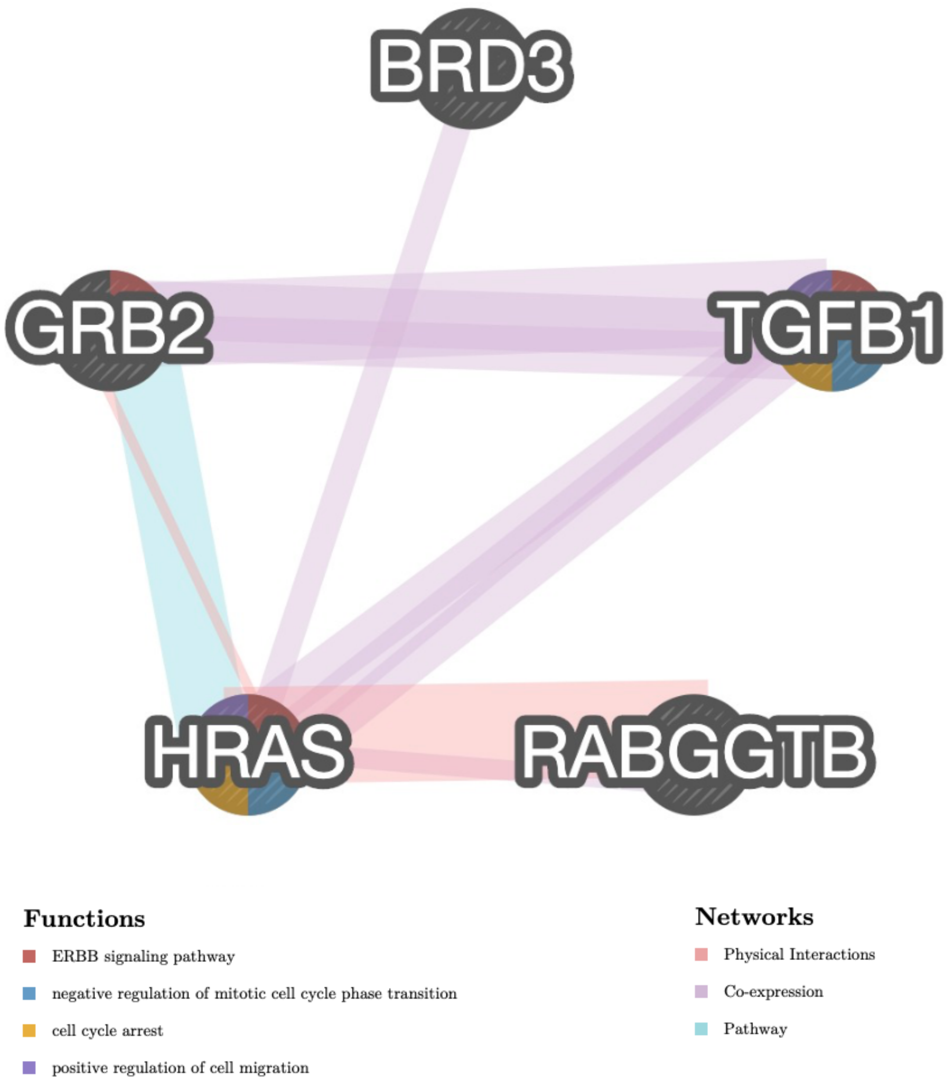

Supplement: Supplementary file 1 [file ijms-21-09061-s001.pdf]
